# Defensive Vocalizations and Motor Asymmetry Triggered by Disinhibition of the Periaqueductal Gray in Non-human Primates

**DOI:** 10.3389/fnins.2017.00163

**Published:** 2017-03-29

**Authors:** Patrick A. Forcelli, Hannah F. Waguespack, Ludise Malkova

**Affiliations:** ^1^Department of Pharmacology and Physiology, Georgetown UniversityWashington, DC, USA; ^2^Interdisciplinary Program in Neuroscience, Georgetown UniversityWashington, DC, USA; ^3^Department of Neuroscience, Georgetown UniversityWashington, DC, USA

**Keywords:** defensive behavior, macaque, PTSD, primate, GABA-A, bicuculline

## Abstract

Rapid and reflexive responses to threats are present across phylogeny. The neural circuitry mediating reflexive defense reactions has been well-characterized in a variety of species, for example, in rodents and cats, the detection of and species-typical response to threats is mediated by a network of structures including the midbrain tectum (deep and intermediate layers of the superior colliculus [DLSC]), periaqueductal gray (PAG), and forebrain structures such as the amygdala and hypothalamus. However, relatively little is known about the functional architecture of defense circuitry in primates. We have previously reported that pharmacological activation of the DLSC evokes locomotor asymmetry, defense-associated vocalizations, cowering behavior, escape responses, and attack of inanimate objects (Holmes et al., 2012; DesJardin et al., 2013; Forcelli et al., 2016). Here, we sought to determine if pharmacological activation of the PAG would induce a similar profile of responses. We activated the PAG in three awake, behaving macaques by microinfusion of GABA-A receptor antagonist, bicuculline methiodide. Activation of PAG evoked defense-associated vocalizations and postural/locomotor asymmetry, but not motor defense responses (e.g., cowering, escape behavior). These data suggest a partial dissociation between the role of the PAG and the DLSC in the defense network of macaques, but a general conservation of the role of PAG in defense responses across species.

## Introduction

Rapid and reflexive responses to threats are present across phylogeny. These defensive reactions are manifest in species-typical ways; for example, rodents display freezing both to looming stimuli and to unconditioned fear-evoking stimuli, such as fox odor (Cattarelli and Chanel, 1979; Wallace and Rosen, 2000; Yilmaz and Meister, 2013; Shang et al., 2015; De Franceschi et al., 2016). By contrast, primates display alarm calls, avoidance and escape behaviors toward unconditioned fear-provoking stimuli, such as snakes (Izquierdo and Murray, 2004; Shibasaki et al., 2014; Weiss et al., 2015; Kawai and Koda, 2016). In humans, midbrain regions (encompassing the periaquaductal gray and the deep/intermediate layers of the superior colliculus) parametricaly encoded proximity of threating stimuli (Mobbs et al., 2010) and looming threats (Coker-Appiah et al., 2013). The brain networks subserving these behaviors have been well-characterized in rodents and rely critically on the interaction between mid-brain structures (e.g., the superior and inferior colliculi, the periaqueductal gray) and limbic regions (e.g., the amygdala) (Brandão et al., 1994, 2003; Eichenberger et al., 2002; Schenberg et al., 2005; Coimbra et al., 2006; Ullah et al., 2015). However, less is known about the networks controlling defensive responses in primates.

We have recently reported that selective pharmacological activation of the deep and intermediate layers of the superior colliculus (DLSC) evokes a constellation of defensive behaviors (DesJardin et al., 2013). Microinjection of the GABA-A receptor antagonist, bicuculline methiodide, into the DLSC evoked, in a dose-dependent manner, cowering behaviors, explosive escape reactions, alarm vocalizations, and attack of inanimate objects. These behaviors co-occurred with a concurrent reduction in affiliative social interactions when animals were examined in social dyads. Interestingly, transient pharmacological inhibition of the amygdala was able to attenuate some (i.e., cowering) but no other (escape, vocalizations) behaviors evoked by disinhibition of the DLSC (Forcelli et al., 2016). These reflexive behaviors bear striking similarity to some behaviors reported in rodents after activation of the DLSC; for example, rodents likewise display explosive escape behaviors and postural asymmetry following activation of DLSC (Dean et al., 1986; Sahibzada et al., 1986). However, the occurrence of these striking defensive responses in the primate was somewhat surprising given that despite a long history of microinjection into the primate SC, defensive responses had not been previously reported. The fact that these behaviors were not reported (prior to our study) in primates led to the proposal by Redgrave and Dean that emphasized a preferential role of primate DLSC for approach responses rather than defensive responses (Dean et al., 1989). However, our findings indicate that the function of DLSC as a substrate for responding to threats is conserved across species.

Given our finding that the role of DLSC in defensive responses appears to be conserved between rodents and primates, we next sought to examine the role of a sub-adjacent structure, the periaquaductal gray. In rodents, activation of the PAG triggers freezing responses (Bandler et al., 1985a; Krieger and Graeff, 1985). Moreover, in cats, vocalization, defense, and orienting reactions have been reported after PAG activation (Bandler and Carrive, 1988). In the macaque, while vocalizations have been reported, defensive responses have not been reported after PAG activation (Larson and Kistler, 1986). However, anxious temperament in macaques is correlated with increased brain metabolism in PAG as measured by FDG-PET (Fox et al., 2008). Further supporting a role for this structure across species, neuroimaging studies in humans have revealed a topography of PAG activation in response to viewing an aversive image, with large magnitude signal changes seen in the caudal ventromedial and ventrolateral PAG, and the rostral lateral PAG (Satpute et al., 2013). Perhaps most interestingly, electrical stimulation of a medial zone of the dorsolateral tegmentum (including the periaquauctal gray and deep portions of the corpora quadrigemina) in humans has been reported to induce feelings of fright (Nashold et al., 1969).

Thus, based on the above findings, we sought to determine if activation of the lateral PAG in macaques, by microinjection of the GABA-A receptor antagonist, bicuculline methiodide, would elicit defensive responses. We consider the evoked responses in light of our prior findings in the DLSC (Holmes et al., 2012; DesJardin et al., 2013; Forcelli et al., 2016).

## Methods

### Animals

Three pigtail macaques (*Macaca nemestrina*) were used in this study, 1 female (JA) and 2 male (GW, ZK). These animals were raised in the Infant Primate Research Laboratory at the University of Washington Regional Primate Research Facility, in a way similar to that described previously (Novak and Sackett, 1997). At ~6 months of age, the animals were transferred to Georgetown University where all experimental procedures were conducted. Animals were pair-housed within two joined individual cages (size: 61 × 74 × 76 cm each) in a temperature (24°C) and humidity controlled room with a standard 12-h light/dark cycle.

When not performing concurrent cognitive testing, animals were given full feed (Primate Lab Diet, #5049, Purina Mills Inc. International, Brentwood, MD) supplemented with fresh fruit. Water was also available *ad libitum* in the home cage. Care and housing of the monkeys met or exceeded the standards as stated in the Guide for Care and Use of Laboratory Animals (National Research Council U.S., Institute for Laboratory Animal Research U.S., and National Academies Press U.S., 2011), ILAR recommendations and AAALAC accreditation standards. The study was conducted under a protocol approved by the Georgetown University Institutional Animal Care and Use Committee.

The present experiments began after the animals were extensively socialized and behaviorally trained (including chair-training), at approximately 2 years of age. In addition to the experimental procedures described here, all subjects were trained on various cognitive tasks administered at the Wisconsin General Testing Apparatus; the tasks included visual object discrimination, visual delayed non-matching to sample, cross-modal auditory-visual matching task, and reinforcer devaluation. As part of those experiments, some animals received drug infusions in BLA (animals JA, and ZK) (Wellman et al., 2005). Additionally, all of these animals received microinjections in the BLA and/or CeA for two other studies of social behavior (Wellman et al., 2016; Forcelli et al., 2017), and two of the animals also received injections into the DLSC (animals ZK and GW) (DesJardin et al., 2013). For these prior studies, the GABA-A receptor agonist muscimol, the GABA-A receptor antagonist bicuculline methiodide, the NMDA receptor antagonist AP-7 or the AMPA receptor antagonist NBQX were injected into the sites described. As documented by the histological evaluation of all the cases, damage to the amygdala (Wellman et al., 2016, Figure 1); (Forcelli et al., 2017, Figure 1) or to DLSC (DesJardin et al., 2013, Figure 1) due to insertion of the cannula was minimal.

### Surgical implantation of cranial infusion platform and localization of infusion sites

Monkeys were implanted with stereotaxically positioned chronic infusion platforms as we have described extensively elsewhere an (Wellman et al., 2005, 2016; West et al., 2011; Holmes et al., 2012; DesJardin et al., 2013; Dybdal et al., 2013; Forcelli et al., 2014, 2016; Malkova et al., 2015). This platform enabled us to target the periaqueductal gray based on the coordinates assessed by structural magnetic resonance imaging (MRI). Prior to surgery, each monkey received a T1-weighted MRI scan to enable precise placement of the platform. The infusion platform was implanted under anesthesia and aseptic conditions, with postoperative analgesics and antibiotics determined in consultation with the facility veterinarian.

Postoperatively, each monkey received at least one T1-weighted scan with tungsten microelectrodes (FHC, Bowdoinham, ME) placed dorsal to the infusion sites calculated based on the pre-operative MRI. The position of these electrodes, which were visible on the scan, were then used to adjust the final infusion coordinates as needed. Our platform allows for 2 mm resolution in the anteroposterior and mediolateral planes, and sub-mm resolution in the dorsoventral plane.

### Drug solutions and intramesencephalic infusions

The GABA_A_ antagonist bicuculline methiodide (BMI; Sigma-Aldrich) was dissolved in saline and injected at a dose of 2.5–7 nmol in 0.5–1 μl volume, unilaterally. Drug infusions were performed aseptically, while the monkey was seated in a standard primate chair (Crist Instruments, Inc.) with minimal restraint. Infusions were performed using procedures we have previously described (Malkova et al., 2015). A sterile injector cannula was acutely placed into the PAG using the pre-determined coordinates. This 27-gauge cannula was connected, via sterile tubing, to a Hamilton syringe controlled by an infusion pump. The pump was calibrated to deliver solution at a rate of 1 μl / 5 min. After completion of infusion, the cannula was left in place for 1–5 min prior to removal, to minimize drug reflux up the cannula track. The entire infusion procedure lasted 10–15 min. Behavioral observation was initiated within 15 min following an infusion.

### Behavioral assessment

Twenty-four hour prior to each drug infusion, the experimental subject was placed into an observation cage (61 × 74 × 76 cm) and video-taped for 30 min; these “baseline” sessions serve as our control. For observation after drug infusion, animals were again transferred to an observation cage and video-taped for 60 min immediately following the completion of drug infusion. The observation cage was placed in room separate from the normal primate housing room and contained no other animals. Consistent with our prior analysis of defensive behaviors evoked from the DLSC (DesJardin et al., 2013), we identified the peak bin (either 0–15 min or 15–30 min) for each infusion and used this single bin for statistical analysis. This allows for slight differences in positioning of infusions across subjects. We **did not** include later bins, because of potential drug diffusion outside of the structure of interest.

Videotapes were analyzed using the software program The Observer (Noldus Information Technology, Wageningen, Netherlands) according to an ethogram consisting of the behavioral categories we have previously described (Holmes et al., 2012; DesJardin et al., 2013; Forcelli et al., 2016). In addition, we recorded the presence of postural/locomotor asymmetries (quadrupedal circling, head deviation greater than 45 degrees from the midline). A list of operational definitions for the behavioral categories is provided in Table 1. Scores of one observer were used for statistical analysis, however, additional observers were trained to achieve a high level of inter-observer correlation (*r* = 0.9 or better) and analyzed a subset of videotapes.

**Table 1:** **Operational definitions of observed behaviors**.

**Behavior**	**Description**
Defense-associated vocalizations	Calls consisting of barks and screams[this excludes affiliative vocalizations, such as coos]
Cower	Withdrawal to the periphery of the cage in a crouched or recoiled position with an upward directed gaze
Escape	Sudden movement/startle response, typically consisting of moving explosively from one side of the cage to the other
Attack of inanimate objects	Bitting, hitting, or throwing objects such as toys and/or ratting cage bars
Motor/postural asymmetry	Quadrupedal circling, head deviation greater then a45 degree from the midline

*Behaviors that were coded from video-records*.

### Histology

**Figure 1:**
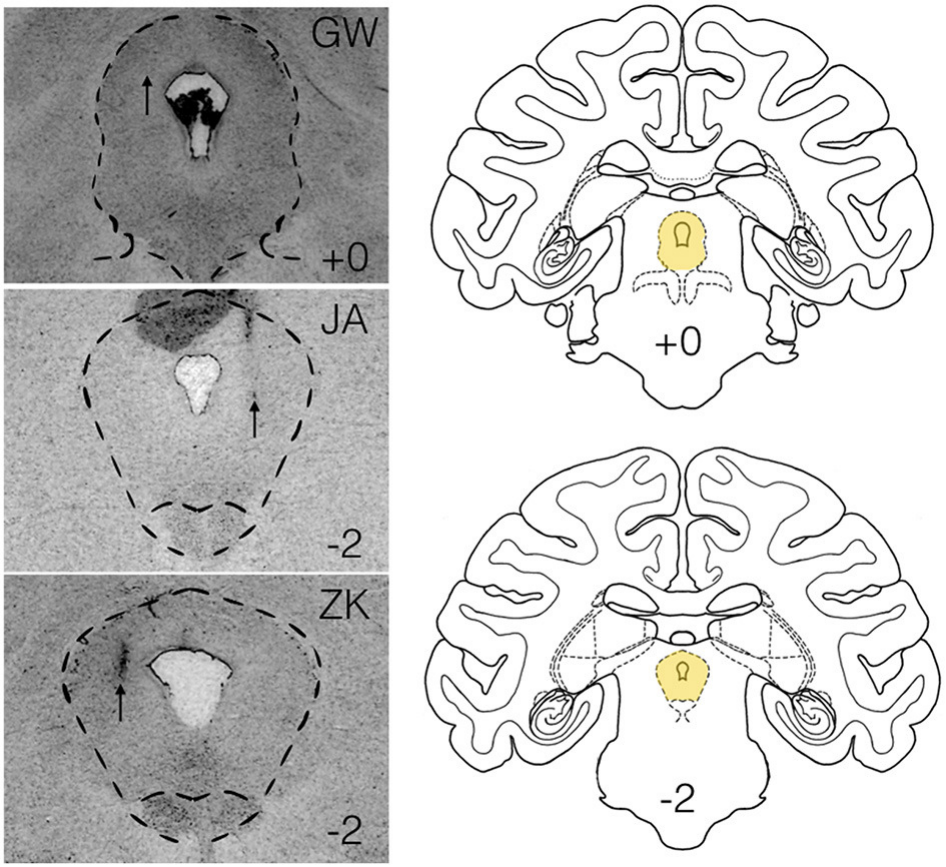
**Histological verification of infusion sites within the lateral PAG**. Panels on the left show infusion sites in the periaqueductal gray of the three subjects (GW, JA, ZK). Outlined region shows the PAG. Atlas panels on the right show the level of infusion sites for GW and JA (top, +0) and ZK (bottom, −2) relative to the intra-aural line. Atlas panels are from the National Institute of Mental Health/Laboratory of Neuropsychology standard Rhesus Macaque atlas.

Animals were perfused and brains processed for localization of infusion sites, as we have previously described (Wellman et al., 2005; Dybdal et al., 2013; Forcelli et al., 2014). Representative photomicrographs for each subject are shown in Figure 1.

### Analysis of vocalizations

We performed a power spectral analysis on three call types from one subject (JA). Calls were isolated from the video/audio-records and transferred to a PC. Sections containing vocalizations in the absence of other room noise were exported as WAV files and imported into LabChart Pro (Version 8.12, AD Instruments). The data were subjected to a fast Fourier transform (1024 bin frequency resolution; Hann window, 93.75% window overlap), and the resulting power spectral density was plotted using LabChart functions.

### Statistical analysis

Data were analyzed using GraphPad Prism (GraphPad Software, Inc, La Jolla, CA). Because defensive behaviors rarely (vocalization, escape; 1 of 6 cases) or never (cowering, attack) occurred under baseline conditions, data were not normally distributed. Thus, a one-tailed Wilcoxon's signed rank test for matched pairs to test the *a priori* hypothesis that drug treatment > baseline. *P*-values < 0.05 were considered to be statistically significant.

## Results

We injected three sites in JA, two sites in GW and one site in ZK. Injection sites and drug doses for each site are shown in Table 2. These infusions were all placed in the lateral/dorsolateral PAG. Representative photomicrographs showing cannula tracks are shown in Figure 1 for the three animals that were available for histology (GW, JA and ZK). Infusions were targeted at the rostro-caudal level of the intra-aural line and the intended infusion zone is also shown in Figure 1. The latency to the onset of behavioral responses was 15 min or less in all subjects; indeed in three of the six cases behavioral responses to bicuculline microinjection were observed immediately at the start of the observation period (JA-58, JA-66, and ZK-70).

**Table 2:** **Infusion sites and drug doses by case**.

**Case**	**Site (depth)**	**Dose (nmol)**
GW-30	1 (39.5)	5
GW-4	1 (38.5)	7
ZK-70	2 (41)	2.5
JA-66	3 (42.5)	2.5
JA-59	3 (42.5)	2.5
JA-58	3 (42)	2.5

*Case indicate the experimental subject (initials GW, ZK, JA) and numbers indicate the drug infusion within each animal (e.g., JA-58 was the 58th injection in subject JA). Site indicates a particular antero-posterior and medio-laterally defined location within each subject (See Figure 1); depth indicates the penetration depth within a particular track. Dose indicates the amount of bicuculline methiodide microinfused in each case*.

**Figure 2:**
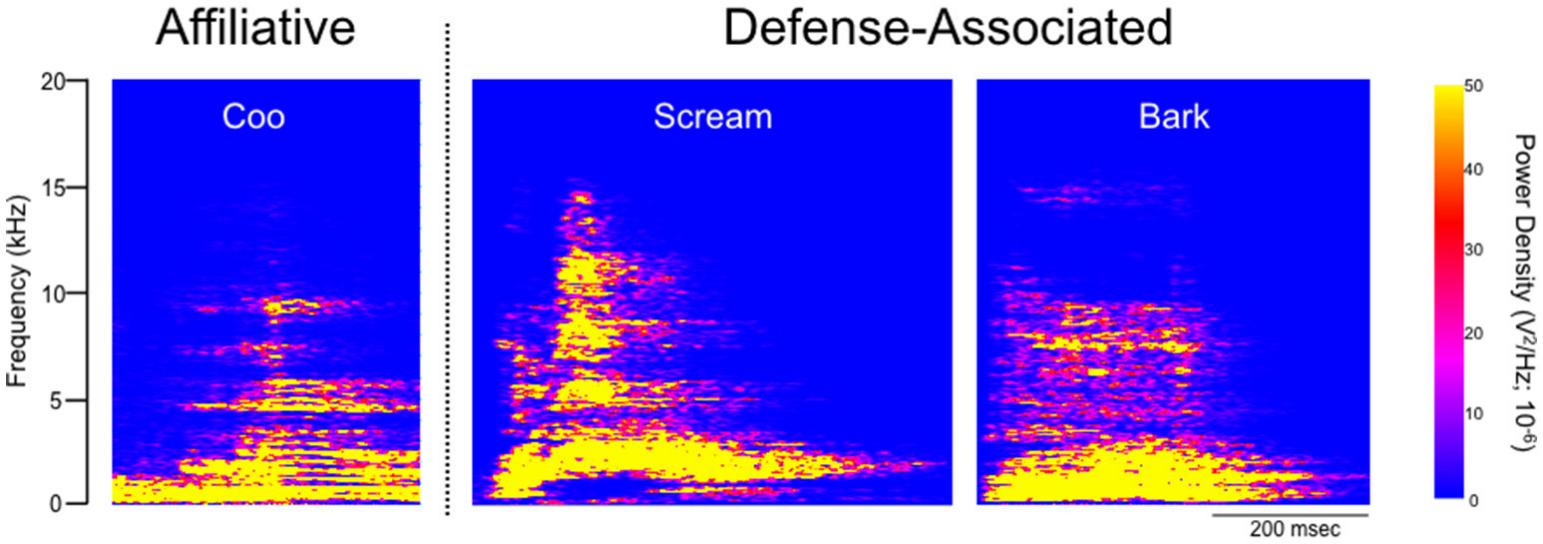
**Spectrographic analysis of macaque calls**. Panels show representative spectrograms for affiliative calls (coo) and defense-associated vocalizations (scream, bark) in a single subject (JA). JA received 2.5 nmol of bicuculline methiodide in PAG.

**Figure 3:**
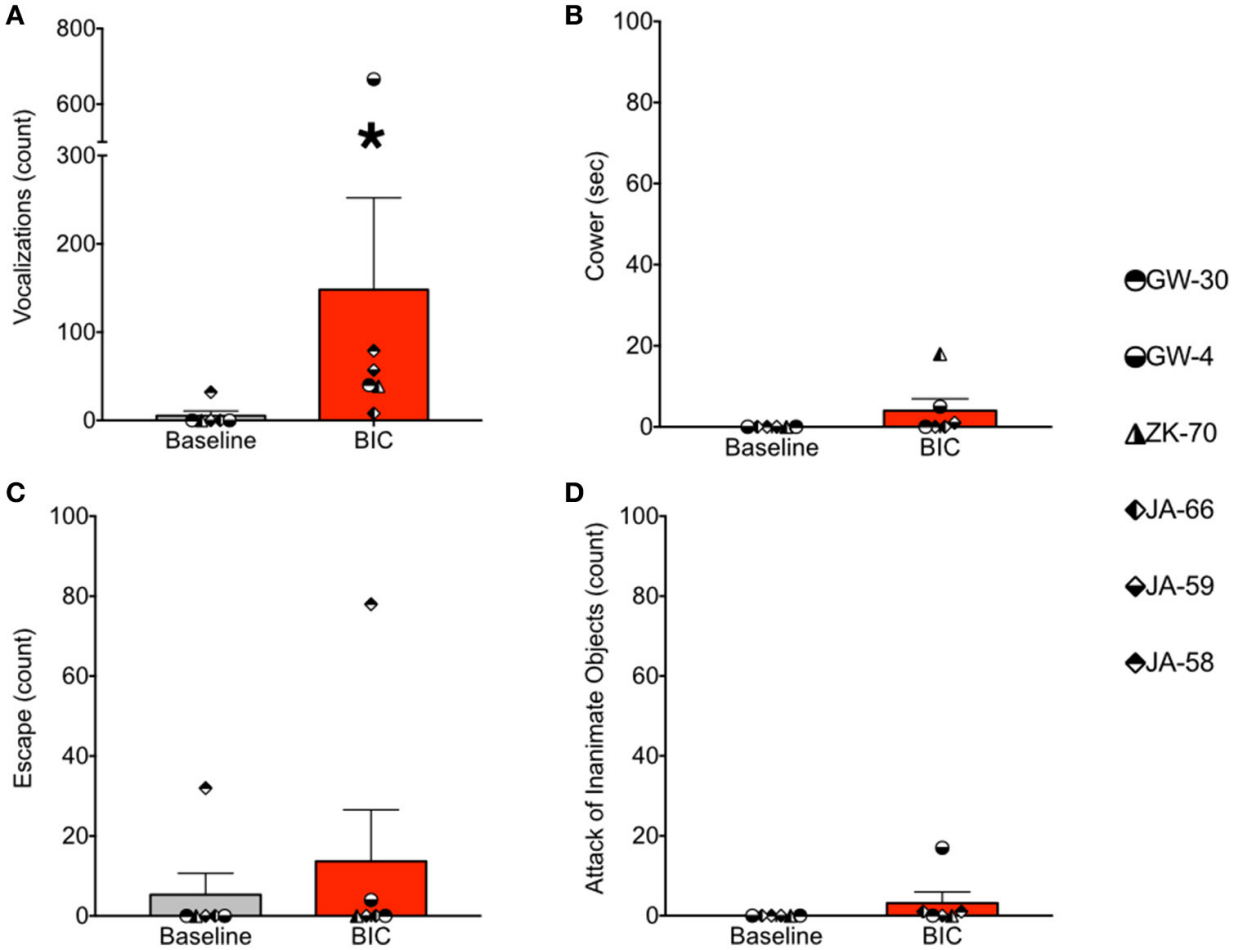
**Bicuculline microinjection in PAG increases defense-associated vocalizations but not motor defense responses. (A)** Number of defense-associated vocalizations over the course of the 15 min observation segment. **(B)** Duration of cowering (seconds) over the course of the 15 min observation segment. **(C)** Number of escape responses over the 15 min observation segment. **(D)** Number of attack of inanimate objects over the 15 min observation segment. Gray bars show mean + standard error for baseline sessions, red bars show mean + standard error for bicuculline microinjection sessions. Symbols represent individual cases. ^*^P < 0.05 (baseline vs. bicuculline infused). JA received 2.5 nmol, ZK received 2.5 nmol, GW-30 received 5 nmol, and GW-4 received 7 nmol of bicuculline methiodide into the PAG.

Under baseline conditions, defense-associated vocalizations were observed in only one session (JA-58) and occurred at a low rate. Figure 2 shows representative spectrograms for defense-associated vocalizations (scream, bark). After microinjection of bicuculline into the PAG, vocalizations emerged in all 6 cases. These vocalizations were characterized by barks and screams, with a mean of 148 vocalizations during the 15-min observation session. These data are shown in Figure 3A. In the one case where vocalizations were observed in the baseline session, the rate of vocalization increased 2.5-fold after bicuculline injection. Coo vocalizations (Figure 2) *never* occurred after activation of PAG; in one case, they were observed under baseline conditions (JA), but abolished after bicuculline infusion into the PAG. Wilcoxon test revealed a significant increase in vocalizations after bicuculline injection in PAG (*W* = 21, *P* = 0.0156).

During the same session, we quantified cowering behavior, escape behaviors, and attack of inanimate objects. Cowering behavior (Figure 3B) was *never* present under baseline conditions. After bicuculline microinjection into the PAG, cowering emerged in three of the six cases, however, it was rare and accounted for less than 2% of the observation session even in the most frequent case. Thus, the occurrence of cowering did not differ significantly between baseline and bicuculline-infused sessions (*W* = 6, *P* = 0.25).

Escape behavior (Figure 3C) was present in one of six baseline sessions (JA-58), and two of six bicuculline-infused sessions (JA-58 and GW-4). Again, the occurrence of this behavior was rare, even when observed. In the case of GW-4, it is worth noting that this was the highest dose of bicuculline delivered in the present study (7 nmol), yet this led to only 4 escape responses during the 15 min observation segment. The occurrence of escape behaviors did not differ between baseline and drug-infused sessions (*W* = 3, *P* = 0.5).

As with cowering behavior, attack of inanimate objects (Figure 3D) *never* occurred under baseline conditions. After bicuculline infusion into the PAG, attack behaviors emerged in three cases (JA-66, JA-58, and GW-4). The occurrence of this behavior, even when observed, was rare in the female subject (1x in JA-66 and JA-58) and more frequent, but still rare in the male subject (GW-4; 17 counts). Again, it is worth noting that case GW-4 received the highest dose of bicuculline in the present study. The occurrence of attack behaviors did not differ significantly between baseline and drug infused sessions (*W* = 6, *P* = 0.25).

**Figure 4:**
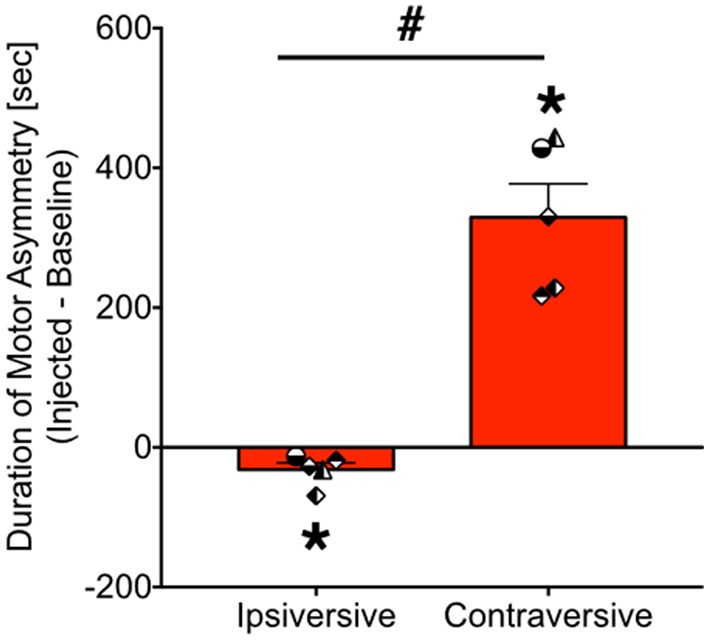
**Bicuculline microinjection in PAG evokes contraversive motor/postural asymmetry**. Bars show the net duration (seconds) of motor asymmetry after bicuculline microinfusion minus the duration of motor asymmetry under baseline conditions [mean + standard error]. Ipsiversive asymmetry indicates motor and posture toward the same side as the injection, whereas contraversive indicates motor and posture directed away from the side of injection. ^*^P < 0.05 as compared to zero (no asymmetry). #*P* < 0.05 contraversive as compared to ipsiversive. Symbols show individual animals, and correspond to the legend in Figure 3. JA received 2.5 nmol, ZK received 2.5 nmol, GW-30 received 5 nmol, and GW-4 received 7 nmol of bicuculline methiodide into the PAG.

As shown in Figure 4, bicuculline microinjection into the PAG resulted in the emergence of striking motor/postural asymmetries. Data are presented as the duration of asymmetry during the injected session minus the duration of asymmetry during the baseline session. Thus, a positive value indicates increased asymmetry in a particular direction, whereas a negative value indicates a reduced asymmetry. A value of zero indicates no change in asymmetry between baseline and infused sessions. These were calculated separately for ipsiversive and contraversive behaviors (relative to the site of PAG injection). Data were analyzed for 5 of the 6 cases (motor behavior for GW-30 was unavailable for analysis). We found that the duration of ipsiversive asymmetry was significantly decreased after infusion of PAG with bicuculline and that the duration of contraversive asymmetry was significantly increased, both with respect to baseline (i.e., a difference score of zero). These effects were revealed by one sample *t*-tests (*Ps* = 0.03 and 0.002, respectively).

## Discussion

Here we report the occurrence of defense-associated vocalizations after activation of the PAG with the GABA-A receptor antagonist, bicuculline methiodide. The volume of tissue activated by our microinjections likely spanned a portion of both the lateral and dorsolateral PAG. Given the relatively short amount of time between our microinjections and the onset of behavioral responses (i.e., 0–15 min), it is likely that 0.5 to 1 mm of tissue was activated. This volume of spread is consistent with prior reports employing gadolinium microinjection from our laboratory (DesJardin et al., 2013; Forcelli et al., 2014) and with the spread of isotopically labeled bicuculline reported by others (Yoshida et al., 1991). The vocalizations evoked from PAG are consistent with prior reports employing electrical stimulation within the PAG (e.g., Larson and Kistler, 1986; Larson, 1991; Larson et al., 1994) of macaques and chemical stimulation of the PAG in the squirrel monkey (Jürgens and Lu, 1993; Lu and Jürgens, 1993). While activation of either the PAG (present study) or DLSC (prior report; DesJardin et al., 2013) induce clear motor/postural asymmetry and defense-associated vocalizations, there was a dissociation between these structures with respect to motor defensive behaviors. While activation of the DLSC induced cowering, escape and attack of inanimate objects, none of these behaviors were observed after activation of PAG.

Microinjection of bicuculline into the DLSC in our prior studies produced cowering, vocalizations, escape responses and attack of inanimate objects. These behaviors were never observed with low-dose bicuculline (e.g., 2.5 nmol), but rather emerged when higher doses were delivered. Indeed, the lowest effective dose for evoking defense responses from the DLSC was 4.6 nmol (DesJardin et al., 2013). Here, with doses of bicuculline as low as 2.5 nmol, vocalizations emerged, and were evoked in all cases. By contrast, after DLSC activation vocalizations were present in only 7 of 9 cases (DesJardin et al., 2013). In the absence of a full dose response for bicuculline microinjection into the PAG, we cannot rule out the possibility that higher doses would have evoked escape behaviors. However, when vocalizations were evoked from DLSC, they always co-occurred with other defense responses, whereas in the PAG, vocalizations occurred without gross motor defense reactions.

### Vocalizations

It has previously been reported in macaques that electrical stimulation in a site within the lateral PAG, comparable to ours, produced inhibition within the spinothalamic tract, perhaps related to analgesia (Gerhart et al., 1984). A similar profile has been reported after GABA antagonist or glycine antagonist injection into lateral PAG of monkeys (Lin et al., 1994). However, because animals were intubated and anesthetized during these experiments, it is perhaps unsurprising that no comment was made regarding the presence or quality of vocalizations or emergence of defensive responses. In the cat, PAG neurons project to the nucleus ambiguus and nucleus retroambiguus (Holstege, 1989); a similar topography is likely in the macaque, in which second order neurons project from nucleus retroambiguus to laryngeal motor neurons (VanderHorst et al., 2001). These connections position the PAG as a likely candidate to mediate defense-related vocalizations. Neurophysiological recordings in macaques have demonstrated that neurons in the lateral PAG burst fire shortly before or during the onset of spontaneous vocalizations and cease firing during or just after a vocalization ends (Larson and Kistler, 1986). Similarly, respiratory-related neurons within the PAG burst during the inspiratory phase of respiration. Of interest, however, these cells have been described to fire during both spontaneous “coo” vocalizations as well as “bark” vocalizations (Larson, 1991). Here, coo vocalizations were *never* evoked after activation of PAG, indeed in the three cases in which affiliative vocalizations (such as coos) were observed under baseline conditions, they were completely abolished after activation of PAG.

The PAG may represent part of a final common pathway mediating defensive vocalizations. For example, defensive-associated hissing responses can be evoked by stimulation of the PAG in cats (Carrive et al., 1987; Bandler and Carrive, 1988; Wang et al., 2002). While similar responses can be evoked from the medial hypothalamus, these responses *require* the PAG: pre-treatment of the PAG with NMDA receptor antagonists abolishes the hypothalamic-evoked hissing responses (Schubert et al., 1996). Similarly, the PAG may play a role in defensive vocalizations evoked from other structures (e.g., the DLSC). It is thus possible that the defense-associated vocalizations we observed after activation of the DLSC, may likewise require the PAG. In support of the idea that these two structures are functionally interconnected, neurons within the PAG are modulated during spontaneous eye saccades in macaques, a behavior closely associated with and requiring the DLSC (Kase et al., 1986). Moreover, the deep layers of the SC project to the lateral PAG in the rat (Beitz, 1982). Whether inhibition of PAG will attenuate SC-evoked vocalizations remains to be tested.

While the nature of vocalizations evoked from the PAG has not previously been characterized in macaques, in squirrel monkeys even lower amounts of bicuculline than those used in the present study have been tested (0.1–1 nmol, threshold dose) (Lu and Jürgens, 1993). Injections of bicuculline into the PAG of squirrel monkeys evoked vocalizations with latencies similar to those that we report here (seconds to tens of minutes), with responses lasting for minutes to hours (Lu and Jürgens, 1993). Sites throughout the dorsoventral extent of the lateral PAG produced vocalizations. Common vocalizations included “peeps” which are alert calls, squeaks, which are frustration calls, and shrieks, which indicate defensive threat (Lu and Jürgens, 1993). Trill calls, which are primarily positive and emitted in response to pleasurable events in squirrel monkeys rarely occurred after PAG activation. Indeed, trills were seen only in 2 of 28 cases following GABA antagonist injection (Lu and Jürgens, 1993). Interestingly, in a subset of sites, the investigators injected in the DLSC, rather than the PAG. As is our experience in macaques, injections into the DLSC also evoked vocalizations.

In the cat, electrical stimulation, or injection of exictatory amino acids into the PAG has also been associated with defense-related vocalizations. For example, stimulation of the lateral PAG (akin to the site we stimulated in the monkey) triggered hissing and ear retraction. Stimulation of the ventrolateral PAG (deeper than we injected) also evoked howling and growling, piloerection, and back arching. Interestingly, visual and tactile stimulation following kainate microinjection into the PAG elicited attack behaviors in 3 of 4 cats (Bandler and Carrive, 1988). Within the PAG of the cat, some evidence for topograpic organization of vocalization-evoking regions exists. For example, activation of the rostral PAG preferentially evoked hissing and growling, while activation of the posterior PAG preferentially evoked howling behavior. Moreover, while lateral and dorsal sites along the rostrocaudal axis evoked defensive vocalizations including hissing and growling, medial sites evoked meowing, crying and screaming vocalizations (Wang et al., 2002). In almost all cases, these pharmacological activations triggered an increase in mean arterial pressure (Wang et al., 2002). These behaviors do not require the telencephalon, as they are evident in the decerebrate preparation (Carrive et al., 1987). This hypertensive response is considered a key part of defense reactions evoked from the PAG in cats; it has been suggested that this is mediated by direct projections to a region of the ventrolateral medulla, the subretrofacial nucleus (Carrive et al., 1988). In macaques, the ventrolateral medulla likewise receives input from the PAG, and in particular the lateral PAG (in the approximate zone that we injected in the present study) (VanderHorst et al., 2001). Thus, the degree to which our manipulations would likewise result in hypertensive responses, while unstudied, seems plausible.

### Motor responses

In the present study, activation of the lateral PAG produced a strong motor/postural asymmetry: bicuculline increased asymmetry by an average of 11-fold across subjects. Neurons within the lateral PAG project to the so called medial pontomedullary head-movement region, perhaps providing an anatomical substrate for orienting responses to threatening stimuli (Cowie et al., 1994). Our data in macaques are consistent with reports in other species; for example in rats, lateralized defensive reactions have been reported after PAG activation, including a “defensive sideways” posture, characterized by concave body position contralateral to the injection (Depaulis et al., 1989); this is similar to the defensive posture seen following activation of the DLSC (Sahibzada et al., 1986). Similarly, in cats, injections of excitatory amino acids into the lateral PAG evokes escape movements (rearing, pawing) and jumping responses and lateralized posture (circling, head turning). It is further worth noting that sites that evoked vocalization in the cat were preferentially associated with head turning behavior and escapes (Carrive et al., 1988; Zhang et al., 1990). Despite evoking postural asymmetry, we failed to reliably evoke other motor defense responses in the macaque.

Interestingly, in addition to motor defense reactions, vocalizations, and postural asymmetry, immobility responses have been reported in several species after PAG activation. For example, optogenetic activation of the lateral PAG in the rat induces both freezing, and flight behaviors, with higher irradiance needed to evoke flight as compared to freezing behavior (Assareh et al., 2016). Interestingly, these thresholds were lower in the lateral PAG as compared to the ventrolateral PAG. In the guinea pig, tonic immobility responses are differentially modulated by the dorsal and ventral PAG, with activation of the former reducing tonic immobility and activation of the latter increasing it (Leite-Panissi et al., 2003; Ramos Coutinho et al., 2008). A similar pattern has been reported in the cat, where ventral PAG activation triggers increased immobility (Zhang et al., 1990). Here we did not observe increased immobility after lateral PAG activation, however, the degree to which this response might be evoked by activation of other sites within the PAG (e.g., the ventral or ventrolateral PAG) remains to be determined. It is also possible that freezing responses may be of preferential benefit for prey animals (e.g., rodents) as compared to primates.

## Conclusions

Here we have reported that activation of the lateral/dorsolateral PAG evoked striking defense-related vocalizations and postural asymmetry in macaques. Based on data in the rat, cat, squirrel monkey and guinea pig, we hypothesized that we would evoke both defensive responses and vocalizations (Bandler and Carrive, 1988; Carrive et al., 1988; Depaulis et al., 1989; Jürgens and Lu, 1993; Lu and Jürgens, 1993; Ramos Coutinho et al., 2008; Assareh et al., 2016). To our surprise, defense responses were not common after activation of the lateral/dorsolateral PAG in the macaque. These data provide a dissociation between the pattern of defensive responses evoked from the DLSC and the PAG, suggesting an anatomical specialization within the midbrain circuitry controlling fear. While activation of either structure triggered vocalizations and asymmetry, the PAG showed a lower threshold for evoking vocalizations, and only the DLSC triggered appreciable motor defense reactions.

The PAG is structurally and functionally conserved across phylogeny. In primate species, where there is an elaboration of neocortical areas, the contribution of top-down influence over PAG function in defense responses may be of importance. Indeed, the PAG receives input from the neocortex, including dosolateral and orbital frontal regions, and particularly dense input from medial prefrontal regions, including the cingulate cortex (Bandler and McCulloch, 1984; Bandler et al., 1985b; Falconi-Sobrinho et al., 2017). Similarly, premotor cortex sends extensive projections to the PAG, and in particular the lateral PAG (An et al., 1998). While direct activation of the PAG (e.g., by bicuculline microinfusion) would bypass any top-down control, in the normally behaving animal, a role for high-level cortical input must be considered.

## Author contributions

PF designed the study, conducted experiments, analyzed data, and wrote the manuscript. HW analyzed data and wrote the manuscript. LM designed the study, conducted experiments, analyzed data, and wrote the manuscript.

## Funding

The study was supported in part by R01 MH084069 (LM), R01 MH082364 (LM), K02HD042269 (LM), Cure Autism Now (now Autism speaks; LM), National Alliance for Autism Research (NAAR; now Autism speaks; LM), and KL2TR001432 (PF).

### Conflict of interest statement

The authors declare that the research was conducted in the absence of any commercial or financial relationships that could be construed as a potential conflict of interest.

## Abbreviations

DLSC: deep and intermediate layers of the superior colliculus
PAG: periaqueductal gray
BIC: bicuculline methiodide.
